# Assessment of regional and global left ventricular function with electromechanical mapping: validation against MRI. A PRECISE substudy

**DOI:** 10.1186/1532-429X-17-S1-P143

**Published:** 2015-02-03

**Authors:** Esther Pérez-David, Ricardo Sanz-Ruiz, Raquel Yotti, Javier Bermejo, Maria Eugenia Fernandez Santos, Fernando Sarnago Cebada, Jaime Elizaga Corrales, Francisco Fernandez Avilés

**Affiliations:** Cardiology, Hospital Gregorio Marañón, Madrid, Spain

## Background

Electromechanical mapping (EMM) of the left ventricle (LV) provides voltage and contractility information of the LV (based on linear local shortening, LLS). Although EMM parameters show excellent correlation with viability data by MRI, little information is available regarding performance of EMM for assessment of regional and global LV function. The purposes of our study were: a) to study the relationship between EMM (LLS) and wall motion by MRI in all segments and b) to correlate LV volumes and LVEF with both techniques.

## Methods

The PRECISE clinical trial of freshly isolated adipose-derived stem cells for angiogenesis included pts with chronic stable CAD, reversible perfusion defects detectable by SPECT and not amenable for revascularization. This study was a post-hoc analysis of the baseline and 6-month follow-up data of EMM and MRI parameters corresponding to the 22 p of the PRECISE trial that were enrolled in our center. EMM was performed with the NOGA XP system. MRI studies were performed with a 1.5T scanner (Philips Intera®, The Netherlands) and included cine imaging, first-pass perfusion and late enhancement. EMM and MRI were performed and analyzed by independent observers, blinded to the results of the other technique. MRI variables included: regional wall motion score (normal=1; hypokinetic=2; akinetic=3), LV volumes and LVEF. EMM variables included LLS (%) for every segment, LV volumes and LVEF.

## Results

Segments with better WMS by MRI had significantly higher values of LLS parameters (Figure, left). Regarding global left ventricular function parameters, agreement between NOGA XP and MRI ventricular parameters was good (Figure: center and right graphs): ICC =0.85 ( IC 95% 0.72-0.92), r=0.86 (IC 95% 0.79-0.93) and r=0.66 (IC 95% 0.43-0.81) for LVEDV, LVESV and LVEF respectively. Volumes were slightly underestimated with NOGA compared to MRI (mean difference: 39±26 cc for LVEDV and 19±23 cc for LVESV).

**Figure 1:**
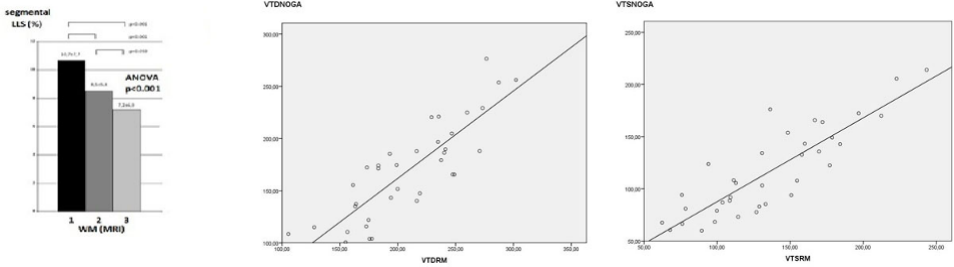
Figure 1

## Conclusions

We show for the first time a good agreement in terms of regional wall motion, LV volumes and ejection fraction between EMM and cardiac MRI.

## Funding

No funding to report.

